# 1764. Collaboration between Public Health and Healthcare Partners to Attack the Rising Menace of *Candida auris*: The Michigan Experience

**DOI:** 10.1093/ofid/ofad500.1595

**Published:** 2023-11-27

**Authors:** Brenda M Brennan, Sara E McNamara, Elisia Stier, Anurag Malani, Jason M Pogue, Adam M Ressler, Seth Eckel, Joseph Coyle, Natasha Bagdasarian

**Affiliations:** Michigan Department of Health and Human Services, Lansing, Michigan; State of Michigan, Lansing, Michigan; Michigan Department of Health and Human Services, Lansing, Michigan; Trinity Health Michigan, Ann Arbor, Michigan; University of Michigan, College of Pharmacy, Ann Arbor, Michigan; University of Michigan School of Public Health, Ann Arbor, Michigan; Michigan Department of Health and Human Services, Lansing, Michigan; Michigan Department of Health and Human Services, Lansing, Michigan; MDHHS, Lansing, Michigan

## Abstract

**Background:**

*Candida auris* is an emerging fungal pathogen with high rates of morbidity and mortality. In May 2021, the Michigan Department of Health and Human Services (MDHHS), detected its first case of *Candida auris* and has since seen a rapid increase in southeast MI. There is currently no *C. auris* screening guidance developed for states. MDHHS Surveillance for Healthcare Associated and Resistant Pathogens (SHARP) Unit convened a multidisciplinary group from acute care facilities to collaborate on the development of guidance to rapidly assess, screen, and isolate patients entering healthcare facilities in efforts to slow the spread of *C. auris.*

**Methods:**

The MDHHS SHARP Unit invited individuals representing infection prevention, infectious diseases, antimicrobial stewardship/pharmacy, lab, and epidemiology to participate. The purpose was to engage healthcare partners by providing updates on epidemiology, surveillance, antifungal susceptibilities, lab capacity, and containment/prevention activities in the state. Facilities were able to share best-practices and collaborate with public health to develop pragmatic recommendations that could be implemented within facilities across the region.

**Results:**

Approximately 60 attendees representing MDHHS and 6 large healthcare systems, providing 63% of patient care in MI, met twice in early 2023 to draft *C. auris* screening guidance that can be customized to the specific facility. The document walks through how to develop a surveillance plan and includes guidance definitions, a Screening Assessment Flowchart, a Screening Assessment Checklist, and an Epidemiologically-linked Health Care Contact Screening Flowchart. The guidance went through an iterative process with feedback from healthcare partners.

**Figure:**
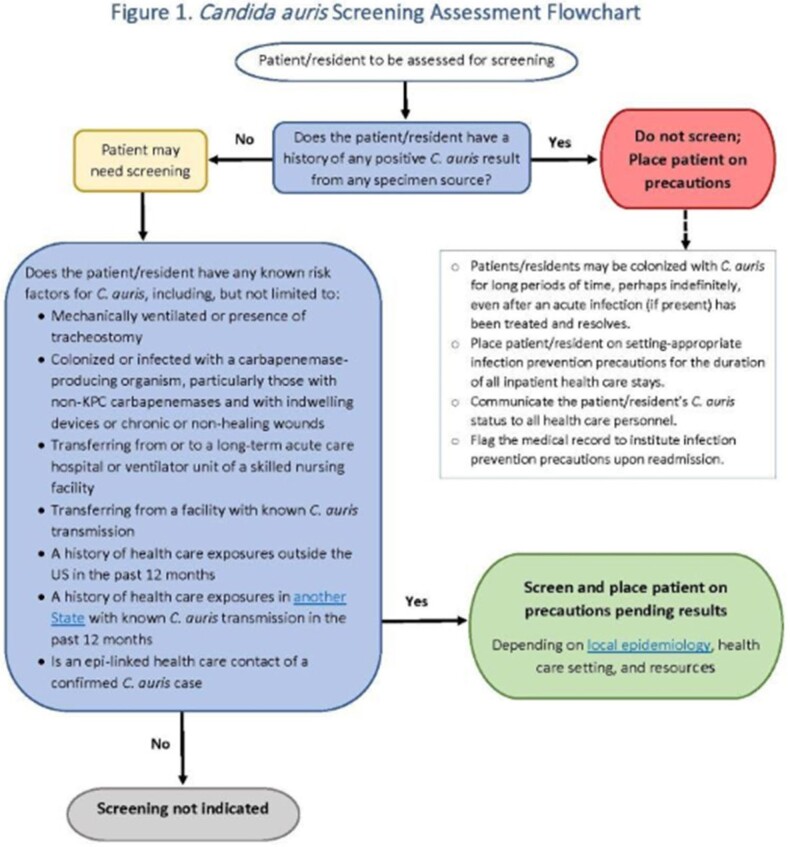

**Figure:**
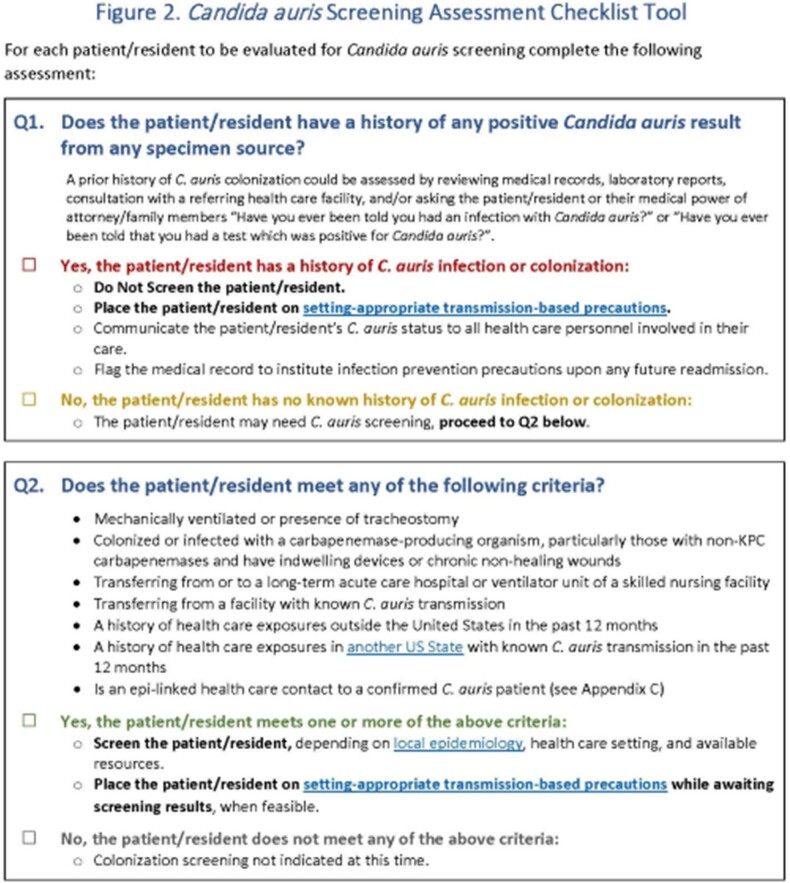

**Figure:**
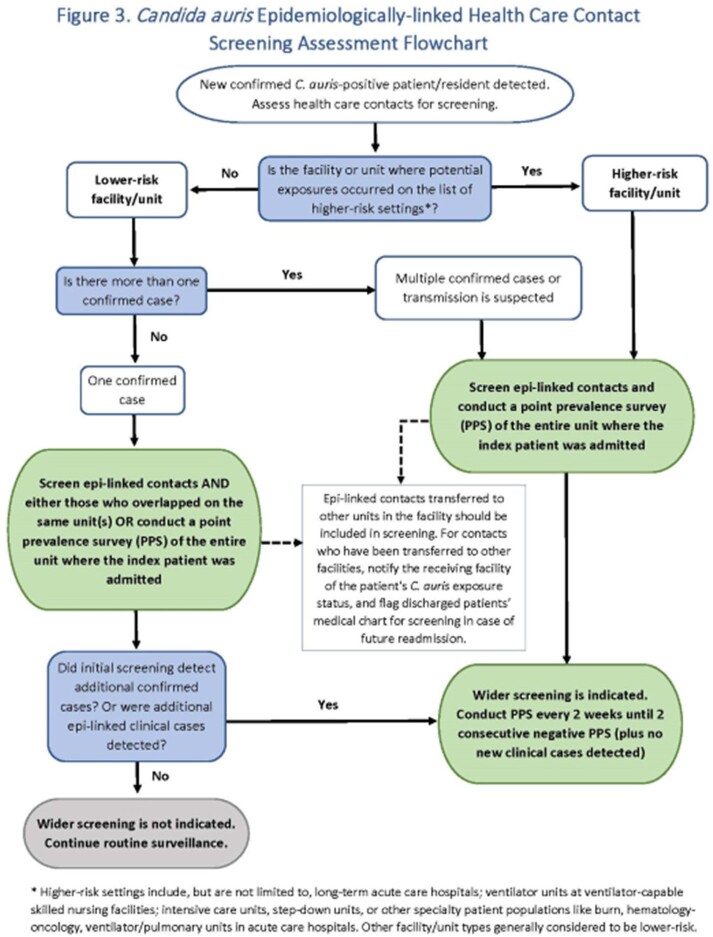

**Conclusion:**

MDHHS collaborated with healthcare partners to develop *C. auris* screening guidance that is evidence-based, easy-to-reference, and adaptable across facility-types. Previous regional approaches, such as the Michigan CRE Surveillance and Prevention Initiative, have proven to be substantially more effective at stemming the spread of multidrug resistant organisms. Michigan will continue these successful collaborations and apply this approach for future emerging pathogens.

**Disclosures:**

**jason M. Pogue, PharmD**, AbbVie: Advisor/Consultant|Entasis: Advisor/Consultant|Ferring: Advisor/Consultant|GSK: Advisor/Consultant|Merck: Advisor/Consultant|Merck: Grant/Research Support|Qpex: Advisor/Consultant|Shionogi: Advisor/Consultant

